# Neuraminidase Inhibitory Activity and Constituent Characterization of *Fagopyrum dibotrys*

**DOI:** 10.3390/molecules22111998

**Published:** 2017-11-18

**Authors:** Xiang Zhang, Yu Cao, Jinhua Li, Ailin Liu, Haibo Liu, Linfang Huang

**Affiliations:** 1 Institute of Medicinal Plant Development, Peking Union Medical College & Chinese Academy of Medical Sciences, Beijing 100193, China; 18364166427@163.com (X.Z.); caoyu0709@139.com (Y.C.); jinhuali108@126.com (J.L.); 2 Institute of Materia Medica, Chinese Academy of Medical Sciences & Peking Union Medical College, Beijing 100050, China; liuailin@imm.ac.cn

**Keywords:** *Fagopyrum dibotrys*, NA inhibitory activity, UHPLC-Q-Exactive, molecular docking, chemical constituents

## Abstract

This study aimed to identify a new biological activity of the widely distributed species *Fagopyrum dibotrys*. Four *F. dibotrys* extracts (ethyl acetate (EA), petroleum ether (P), ethanol (E), and water (W)) were explored for their anti-neuraminidase (NA) activity. A total of 32 compounds were identified using UHPLC-Q-Exactive Orbitrap HRMS in the EA extract, which had the best NA inhibitory effects. We used the docking data for supporting compounds’ anti-neuraminidase activity. Among them, five compounds including one flavonoid, three organic acids, and one glucoside were discovered for the first time in *F. dibotrys*. Docking studies and NA activity assay revealed the remarkable NA inhibitory activity of eight components in EA extract, especially rutin, hesperidin, procyanidin B_2_, and quercitrin. Therefore, *F. dibotrys* could be used to develop anti-influenza drugs.

## 1. Introduction

*Fagopyrum dibotrys* is an important crude drug and functional food that has been widely used in traditional Chinese medicine (TCM) [1]. It mainly grows in China, Vietnam, India, and Thailand [2]. The herb, which has been recorded in the Chinese Pharmacopoeia since 1977, is a functional food and has been approved by National Health and Family Planning Commission of the People’s Republic of China (NHFPC). It also called *Fagopyrum cumosum* (Trev.) Meisn, whose rhizome was considered as folk medicine for clearing away heat and toxic materials, expelling pus and removing blood stasis [3,4,5]. Until recently, some biological activities of *F. dibotrys*, including anti-oxidant, anti-tumor, anti-inflammatory, anti-bacterial [6,7,8], and so on have been reported, but anti-influenza activity is unknown.

**Figure 1:**
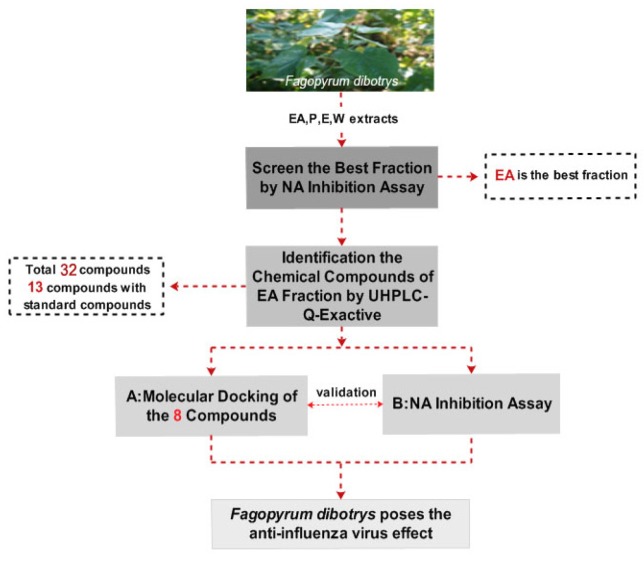
The flowchart of the paper.

The Q-Exactive system with the its high resolving power performance easily combines with ultra-high performance liquid chromatography (UHPLC). UHPLC-Q-Exactive Orbitrap high resolution mass spectrometry (HRMS) can rapidly detect hundreds of components in complex compound mixtures from plants with short running times [9]. Similarly, molecular docking is a reliable and efficient tool for discovering or designing novel drugs used to screen biological chemical compounds [10,11,12,13]. In the present work (see Figure 1 for a flowchart of the protocol used), four *F. dibotrys* extracts (ethyl acetate (EA), petroleum ether (P), ethanol (E), and water (W)) were explored in a neuraminidase (NA) activity assay, with the components of the best extract being further identified by UHPLC-Q-Exactive Orbitrap HRMS. To further validate the NA inhibitory activity of *F. dibotrys*, eight components were docked to the NA inhibition target using molecular docking technology and tested in NA inhibition experiments. Based on the theory that NA is an important target to screen the anti-influenza virus drugs due to the fact it has the essential enzyme activity for virus replication and NA inhibitors are recognized as an effective drugs against influenza A and B by the World Health Organization [14], this research will provide a new direction and basic information for novel anti-influenza virus drug discovery.

## 2. Result and Discussion

### 2.1. Influenza Virus Neuraminidase (NA) Activity Assay of Four Extracts

**Figure 2:**
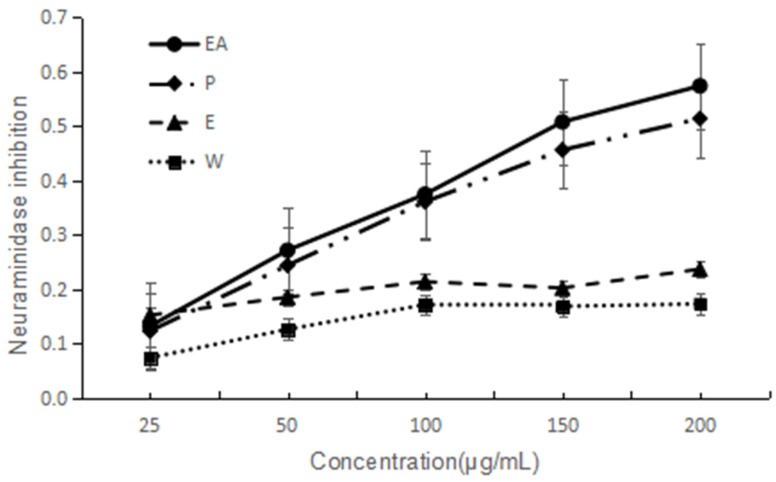
NA inhibitory activity of four extracts of *F. dibotrys*. EA: ethyl acetate fraction; P: petroleum ether fraction; E: ethanol fraction; W: water fraction.

NA has been considered as an important target to screen anti-influenza virus drugs. NA inhibitory activities of the four extracts are shown in Figure 2. They all indicated dose-dependent activity. The lower IC_50_ value implies a higher activity. Based on the IC_50_ values, two extracts are good and EA is the best one. The IC_50_ of EA and P are 149 and 182 µg/mL, respectively. The activity order is EA > P > E > W.

### 2.2. Identification of Chemical Compositions of EA Extract by UHPLC-Q-Exactive

**Figure 3:**
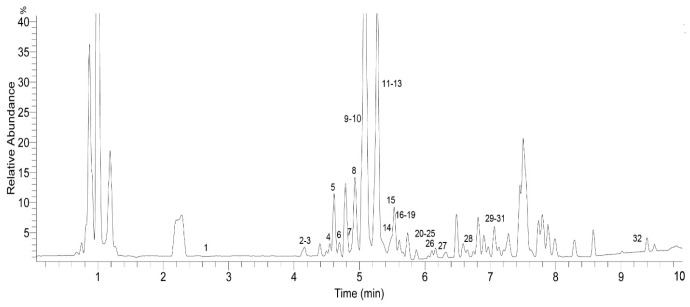
Base peak chromatogram (BPC) of the ethyl acetate fraction (EA) of *F. dibotrys* (negative ion mode).

**Figure 4:**
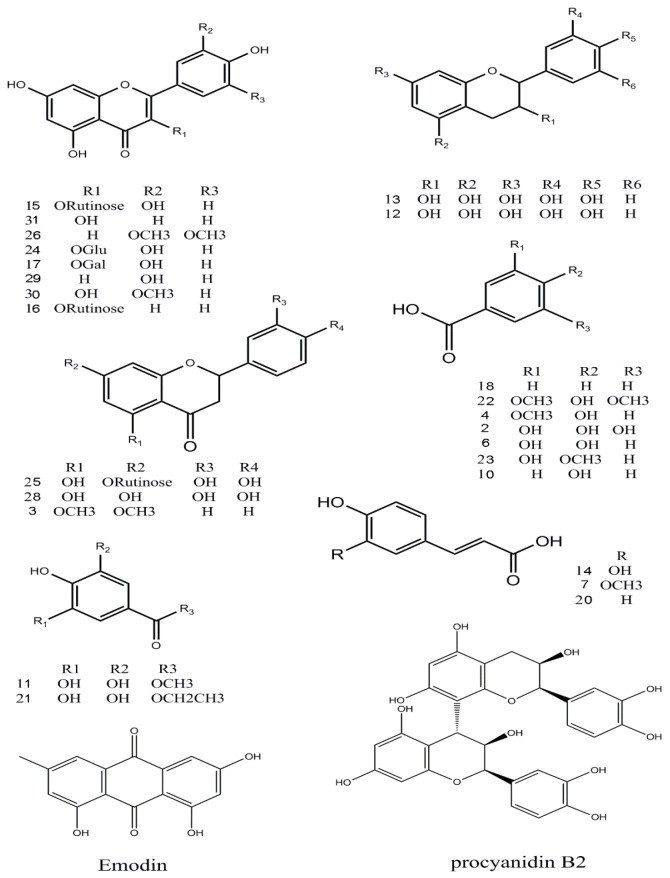
Chemical structures of the main identified compounds in the EA extract from *F. dibotrys*.

The identification of the chemical components of the *F. dibotrys* EA extract was investigated using UHPLC-Q-Exactive HRMS. Based on the retention time, standard substances, molecular ions, and major fragments were observed in MS spectra, followed by a search of an online database (METLIN) and literature reports, wherein 32 chemical structures were tentatively identified and were definitely classified into four groups: flavonoids, tannins, organic acids, and others. Figure 3 and Figure 4 show the EA extract base peak chromatogram (BPC) and the chemical structures of the main identified compounds, respectively. The identified compounds, retention time, molecular formula, exact mass, experimental mass, the major fragments, and references are listed in Table 1.

#### 2.2.1. Flavonoids

Thirteen flavonoid components were identified [15,16,17], and among them six peaks (12, 13, 15, 24, 25 and 28) were matched with standard compounds. Peaks 12 and 13, a pair of isomers with a *m*/*z* 289 molecular ion and a fragment ion of *m*/*z* 245 [M − H − CO_2_]^−^, was proposed to be (−)epicatechin and (+)-catechin, respectively. Peaks 29 and 31, both with a *m*/*z* 285 molecular ion [M − H]^−^, were identified as luteolin and kaempferol, respectively, from a comparison with an online database (METLIN) and literature data. Peak 3 was tentatively identified as 5,7-dimethoxyflavanone for the first time.

#### 2.2.2. Tannins

The only tannin in this study, peak 9, with fragment ions of *m/z* 577 and 289 was proposed to be procyanidin B_2_ which was conformed using a standard.

#### 2.2.3. Organic Acids

Twelve organic acid compounds were detected in the EA extract [3,18,19]. Among them, three peaks (2, 6, 14) were identified by comparison with standard compounds. Peak 10, with a molecular ion [M − H]^−^ at *m/z* 137, and a fragment at *m*/*z* 93 identified as [M − H − CO_2_]^−^ was proposed to be 4-hydroxybenzoic acid. Peaks 4, 8, and 23 were identified as vanillic acid, chlorogenic acid, and 3-hydroxy-4-methoxybenzoic acid, respectively. Peak 8 has a molecular ion at *m*/*z* 353, and the fragment ion of *m*/*z* 191 (M_2_) can be obtained when the ester bond is broken. The ions at 173 *m*/*z* and 154 were proposed to be [M_2_ − H − H_2_O]^−^ and [M_2_ − H − 2H_2_O]^−^; these fragments were in accordance with literature reports. Peaks 1 and 7 were identified as succincic acid and ferulic acid. Peaks 14 and 18 were identified as caffeic acid and benzoic acid, respectively, with corresponding fragment ions at *m*/*z* 135 [M − H − CO_2_]^−^ and *m*/*z* 77 [M − H − CO_2_]^−^, from a comparison with an online database (METLIN) and literature data.

#### 2.2.4. Other Compounds

Peak 11 was proposed to be 4-*o*-methyl-gallate, whose fragment ions were *m*/*z* 169 [M − H − CH_3_ − CO_2_]^−^, *m*/*z* 125 [M − CH_3_ − CO_2_]^−^ [20]. Peak 21 had a molecular ion at *m*/*z* 197, and its fragment ions at *m*/*z* 169 and 125 were identified as [ethyl gallate − CH_3_CH_2_]^−^, and [ethyl gallate − CH_3_CH_2_ − CO_2_]^−^, respectively. The three peaks 11, 19, 21 were confirmed using standard compounds and are consistent with previous reports [21].

Peak 5 was identified as glucosyringic acid, with a *m*/*z* 359 molecular ion; the fragment ion was [M − H − Glu]^−^. The compound protocatechuic acid methyl ester at 6.23 min, showed *m*/*z* 167 and 149 [M − H − H_2_O]^−^ ions. Peak 32, the last compound, was proposed to be emodin with a fragment ion at *m*/*z* 225 [M − H − CO_2_]^−^.

This, a total of 32 compounds, including 13 flavonoids, one tannin, 12 organic acids, and others such as 4-*o*-methyl-gallate, glucosyringic acid, ethyl gallate, ellagic acid, protocatechuic acid methyl ester, and so on, were tentatively identified. Five compounds, including 5,7-dimethoxyflavanone, vanillic acid, 3-hydroxy-4-methoxybenzoic acid, ellagic acid, and glucosyringic acid, were found for the first time in *F. dibotrys*. Some of the same compounds, such as quercitrin, kaempferol, -epicatechin, protocatechuic acid, and chlorogenic acid, were previously reported in the genus *Fagopyrum* (*Fagopyrums tataricum* and *Fagopyrums esculentum*) [3].

### 2.3. In Silico Docking of Eight Chemical Compounds

**Figure 5:**
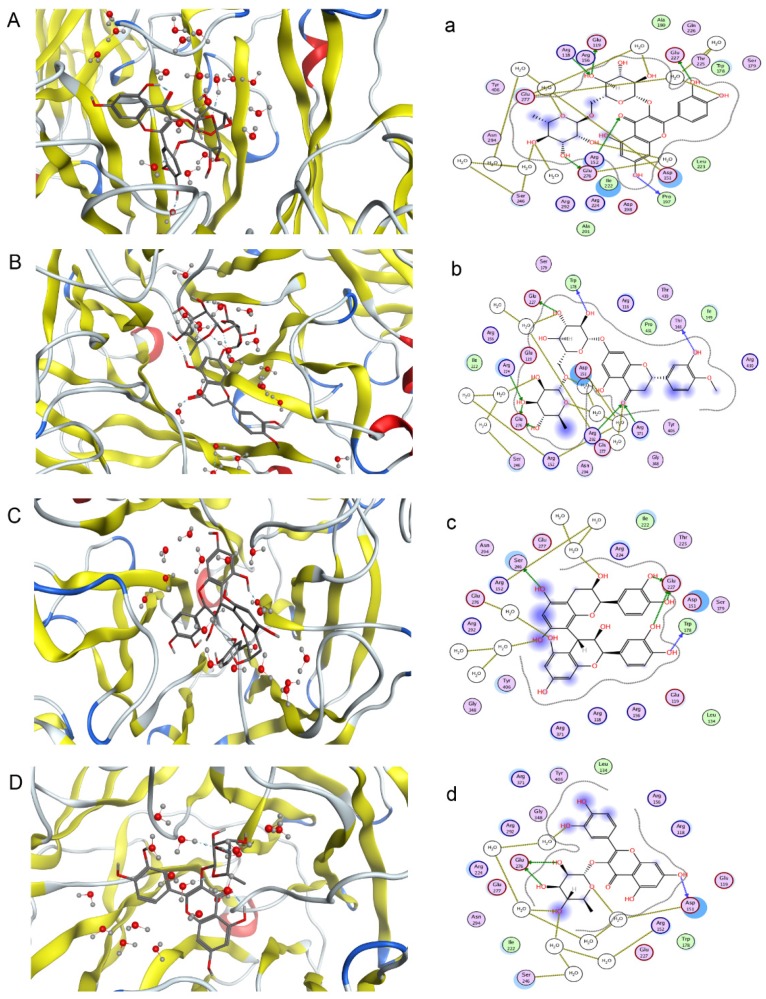
Predicted binding mode of four compounds with NA in three-dimensions (3D) and two-dimensions (2D) (**A**–**D**) rutin, hesperidin, procyanidin B2 and quercitrin with three-dimensional structure; (**a**–**d**) rutin, hesperidin, procyanidin B2 and quercitrin with two-dimensional structure).

One objective of this study was to compare its results and those of pharmacological neuraminidase assay experiments to confirm the activities of compounds in *F. dibotrys*. Eight main chemical compounds, such as procyanidin B_2_, rutin, hesperidin, quercitrin, eriodictyol, caffeic acid, (−)epicatechin, and (+)-catechin, were selected for this part of the study. They were docked to 3TI3 that was selected based on the PharmaDB target database. Based on free energy, a higher absolute value implies a higher biological activity. Four compounds had good NA inhibitory activity, and procyanidin B_2_ was the best one. The indicated docking results were procyanidin B_2_ > rutin > hesperidin> quercitrin > eriodictyol > caffeic acid > (−)epicatechin > catechin. Figure 5 shows the first four compounds’ binding mode with NA. The absolute values of INT can be seen from the Table S1 in supplementary.

### 2.4. Neuraminidase (NA) Experiment of the Main Chemical Compounds

**Figure 6:**
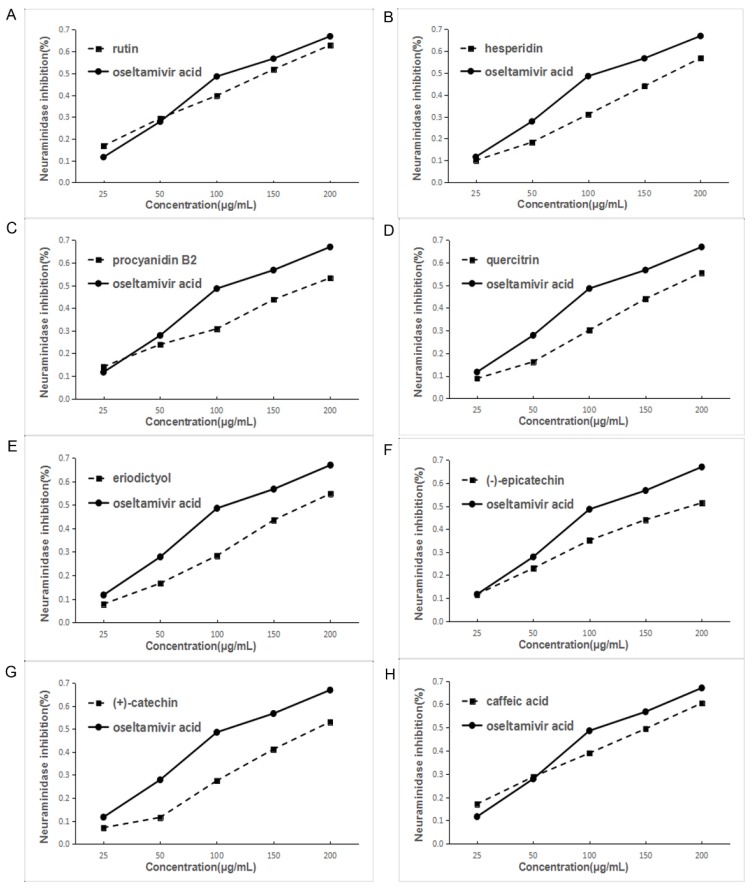
Inhibition of NA activity by various components of EA extract of F. dibotrys. ((**A**), rutin; (**B**), hesperidin; (**C**), procyanidin B_2_; (**D**), quercitrin; (**E**), eriodictyol; (**F**), (−)-epicatechin; (**G**), (+)-catechin; (**H**), caffeic acid).

Based on the above results of the influenza virus NA activity assay of the EA extract and eight main chemical compounds, NA experiments were conducted with the eight main chemical compounds. Oseltamivir acid was the positive control. Each component has different degrees of activity in the inhibition assay. Among them, rutin had the best effect and the order is rutin > hesperidin > procyanidin B_2_ > quercitrin > eriodictyol > epicatechin > catechin > caffeic acid, which is roughly similar to the in silico docking result. Figure 6 and Table 2 show the inhibition of NA activity and IC_50_ values of all the compounds.

According to the results from the computational approach and neuraminidase (NA) experiments, the first three compounds, rutin, hesperidin, and procyanidin B_2_, indeed have NA inhibitory effects. Based on this conclusion, *F. dibotrys* maybe have an anti-influenza virus effect.

Every year, deaths occur around the world because of the influenza virus, which has the highest morbidity among infectious diseases [22]. At present, the NA inhibitors are the first options in clinical practice against the disease. However, chemical drugs can easily cause side effects and drug-resistant virus. Thus, searching for new resources against the influenza virus is necessary and meaningful. In this paper, we demonstrated for the first time that *F. dibotrys* can cause certain NA inhibitory effects from three aspects (extracts, docking and single compounds). The present work would provide basic information and a new research direction for further further tests in vitro and clinical research on *F. dibotrys*.

## 3. Materials and Methods

### 3.1. Plant Material and Sample Preparation

Samples of *F. dibotrys* were collected in Dali, located in central Yunnan, China, in June 2012. The samples were identified by Professor Linfang Huang, and the voucher specimens (CMPB00359) were deposited in the Herbarium of the Chinese Academy of Medical Science & Peaking Union Medicinal College. Samples of dried *F. dibotrys* root were crushed to a fine powder in a pulverizer. One kg of powder was extracted by infusion with 2.5 L of petroleum ether for 24 h. Then, the residue was extracted with 80% ethanol using countercurrent extraction for three times and 2 h each time. The filtered extracted solutions were concentrated using a rotary evaporator to remove the ethanol solution. The concentrated solution was then successively extracted with ethyl acetate using the liquid-liquid extraction method, and the solvent was removed to obtain a dry form of the ethyl acetate fraction using a rotary evaporator. The residue was evaporated to dryness and extracted with water then evaporated to dryness, and the water extract was obtained. The four extracts were used for NA inhibition assays, and the best one was used for qualitative analysis by UPLC-Q-Exactive. The extract was dissolved in methanol at concentrations (*w*/*v*) of 5 mg/mL and filtered through 0.22 µm nylon micropore membranes before chemical characterization.

### 3.2. Chemicals and Standard Substances

LC-MS grade acetonitrile was purchased from Fisher Scientific (Beijing, China). De-ionized water was purified through a Milli-Q system (Millipore, Bedford, MA, USA). The analytical grade reagents used for extraction were obtained from Beijing Chemical Plant Co. Ltd. (Beijing, China). Gallic acid, ellagic acid, ethyl gallate, methyl-gallate, rutin, epicatechin, procyanidin B2, protocatechuic acid, quercitrin, hesperidin, eriodictyol, and caffeic acid were purchased from Chengdu Must Biotechnology Co. Ltd. (Chengdu, China). Oseltamivir acid was purchased from Medchem Express, LLC (Monmouth Junction, NJ, USA). Neuraminidase Inhibitors Screen Kit (NO. P0309) was purchased from Beyotime Institute of Biotechnology Co. Ltd. (Shanghai, China) and includes 10 mL buffer, 1 mL NA, 1 mL fluorescent substrate, and 1.2 mL Milli-Q water. Ninety six-well microplate reader (3925, Costar Company, Bethesda, MD, USA).

### 3.3. Software and Docking Studies

The protein crystal (PDB ID: 3ti3) was downloaded from the PDB database (http://www.rcsb.org/pdb) and docked using the Molecular Operating Environment (MOE) 2014.09 (Chemical Computing Group Inc., Montreal, QC, Canada).

### 3.4. UPLC-Q-Exactive Analysis

#### 3.4.1. Liquid Chromatography

UHPLC analysis was performed using an Ultimate 3000 system (Dionex, Sunnyvale, CA, USA), connected to an online vacuum degasser, an autosampler, a quaternary pump, and a thermostatted column compartment. The chromatographic separation column was ACQUITY UPLC HSS T3, 2.1 mm × 100 mm, 1.7 µm (Waters, Milford, MA, USA) at 40 °C. The separation conditions contain a gradient elution using acetonitrile as phase A and aqueous formic acid 0.1% (*v*/*v*) as mobile phase B at a flow rate of 0.3 mL/min. The following gradient was applied: 0–1 min, 0% A; 1–10 min, 0–100% A; 10–10.1 min, 100–0% A and 10–10.1 min, 0% A. The injection volume was 2 µL, and the injection temperature was 15 °C.

#### 3.4.2. Mass Spectrometry

Mass spectrometry was executed using a Q Exactive Orbitrap MS system (Thermo Fisher, Waltham, MA, USA) with a heated electrospray ionization source for the ionization of the target compounds under the negative mode. The following are operating parameters: auxiliary gas heater temp, 300 °C; spray voltage, 3.70 KV; capillary temp, 320 °C; auxiliary gas pressure, 10 arb; sheath gas pressure, 30 psi; scan modes, full MS (resolution 70,000), and scan range, 100–1500 *m*/*z*. The data were processed using the Xcalibur software (Thermo).

### 3.5. Neuraminidase (NA) Inhibition Assay

Oseltamivoir acid, four extracts and other eight compounds (all of them were tested as inhibitors) were dissolved in DMSO to give a 1 mg/mL solution. They were then diluted to 100 μg/mL and 200 μg/mL using buffer solution. The experiments were executed in a 96-well microplate reader using the procedure provided in the kit instructions. A reaction mixture containing 70 µL of reaction buffer solution, 10 µL of NA and 10 µL of inhibitors with concentrations of 25, 50, 100, 150 and 200 μg/mL diluted from mother liquor, were added to each well respectively. Every concentration existed in three holes and they were added to 10 µL Mill-Q water for each wells’ buck were 90 µL. Vibration mixing was performed for approximately 1 min and incubation for 2 min at 37 °C, so that the NA and inhibitors react sufficiently. Afterwards, 10 µL of fluorescent substrate was added to produce a total of 100 µL reaction mixture. The whole mixture was completely mixed by vibration for 1 min, and the plate was incubated for 30 min at 37 °C. The fluorescence was read using a microplate spectrophotometer (Molecular Devices, Gemini EM, Shanghai, China) with 322 nm of excitation wavelength and 450 nm of emission wavelength. The experiment was repeated three times. Oseltamivoir acid was used as a positive control. The inhibition (%) was calculated using the formula:

NA Inhibitory activity (%) = [1 − (F_s_ − F_0_)/(F_m_ − F_0_)] × 100%

F_s_ was fluorescence intensity of inhibitors in the presence of the sample, F_0_ was the fluorescence intensity of the blank control and F_m_ was the fluorescence intensity of the negative control. The 50% inhibitory concentration (IC_50_) was analyzed by probit regression in SPSS (version 21, IBM SPSS, Chicago, IL, USA).

### 3.6. Statistical Analysis

NA inhibition assay was conducted in triplicate. Graph Pad Prism 6 (GraphPad, San Diego, CA, USA) and SPSS 21 were used for the statistical analysis of the data.

## 4. Conclusions

The NA inhibitory effect of *F. dibotrys* was revealed for the first time. An advanced UHPLC-Q-Exactive method and a novel computational approach were developed to successfully elucidate the complicated chemical composition of *F. dibotrys*. A total of 32 compounds, including 13 flavonoids, one tannin, 12 organic acids, and six other compounds, were characterized. Among them, five compounds were reported for the first time in *F. dibotrys*. This work showed that UHPLC-Q-Exactive Orbitrap HRMS was a rapid and reliable tool for complex plant chemical component analysis and that *F. dibotrys* had NA inhibitory activity based on molecular docking. Further research is essential to explore the molecular mechanisms. Our laboratory group will further explore these effects through in anti-influenza virus experiments. This study provided scientific evidence and a new direction for future research on improving the resource efficiency of *F. dibotrys*.

## Figures and Tables

**Table 1:** Thirty-two chemical constituents identified in the ethyl acetate fraction (EA) of *F. dibotrys* extracts using UHPLC-Q-Exactive.

Peak	Assigned Identify	Rt (min)	Formula Ions	Calculated (*m*/*z*)	Experimental (*m*/*z*)	Fragment Ions	Reference Sources
1	succinic acid	2.65	C_4_H_6_O_4_	117.01824	117.01784	73	[3]
2	gallic acid	4.13	C_7_H_6_O_5_	169.01315	169.01306	125	[15]
3	5,7-dimethoxyflavanone	4.16	C_17_H_15_O_4_	282.08866	282.08411	268	First report in *F. dibotrys*
4	Vanillic acid	4.56	C_8_H_8_O_4_	167.03389	167.03386	149	First report in *F. dibotrys*
5	glucosyringic acid	4.61	C_15_H_20_O_10_	359.09727	359.09808	197	First report in *F. dibotrys*
6	protocatechuic acid	4.69	C_7_H_6_O_4_	153.01824	153.0181	109	[3]
7	ferulic acid	4.84	C_10_H_10_O_4_	193.04954	193.04948	178	[13]
8	chlorogenic acid	4.93	C_16_H_18_O_9_	353.08671	353.08743	191	[3]
9	procyanidin B_2_	5.08	C_30_H_26_0_12_	577.13405	577.13464	289	[3]
10	4-hydroxybenzoic acid	5.15	C_7_H_6_O_3_	137.02332	137.02303	93	[3]
11	4-*o*-methyl-gallate	5.20	C_8_H_8_O_5_	183.0288	183.02884	169,125	[12]
12	(−)epicatechin	5.27	C_15_H_14_O_6_	289.07066	289.07141	245	[3]
13	(+)-catechin	5.27	C_15_H_14_O_6_	289.07066	289.07141	245	[2]
14	caffeic acid	5.32	C_9_H_8_O_4_	179.03389	179.03378	135	[13]
15	rutin	5.53	C_27_H_30_O_16_	609.14501	609.14612	301	[2,3]
16	kaempferol-3-*o*-rutinoside	5.55	C_27_H_30_O_15_	593.1501	593.15076		[3]
17	hyperoside	5.69	C_21_H_20_O_12_	463.0871	463.08832	301,271	[5]
18	benzoic acid	5.71	C_7_H_6_O_2_	121.02841	121.02805	77	[3]
19	ellagic acid	5.72	C_14_H_6_O_8_	300.99789	300.99881	299.01556	First report in *F. dibotrys*
20	4-Hydroxycinnamic acid	5.82	C_9_H_8_O_3_	163.03897	163.03893	146,119	-
21	ethyl gallate	5.83	C_9_H_10_O_5_	197.04445	197.0446	169,153	[14]
22	Syringic acid	5.84	C_9_H_10_O_5_	197.04445	197.04466	153	[13]
23	3-hydroxy-4-methoxybenzoic acid	5.93	C_8_H_8_O_4_	167.03389	167.03386		First report in *F. dibotrys*
24	quercitrin	5.95	C_21_H_20_O_11_	447.09219	447.09296	301	[2,3]
25	hesperidin	6.00	C_28_H_34_O_15_	609.1814	609.18292	301	[3]
26	3′′,5′-dimethoxy-4′,5,7-trihydroxyflavone	6.06	C_17_H_14_O_7_	329.06558	329.06671		-
27	protocatechuic acid methyl ester	6.23	C_8_H_8_O_4_	167.03389	167.03389	149	[3]
28	eriodictyol	6.79	C_15_H_12_O_6_	287.05501	287.05585		-
29	luteolin	6.92	C_15_H_10_O_6_	285.03936	285.04044	133	[3]
30	isorhamnetin	7.02	C_16_H_12_O_7_	315.04993	315.05099	300	-
31	kaempferol	7.13	C_15_H_10_O_6_	285.03936	285.04059	255	[3]
32	emodin	9.22	C_15_H_10_O_5_	269.04445	269.04535	241	[2]

**Table 2:** The IC_50_ values of 8 main chemical compounds in *F. dibotrys* on neuraminidase (NA) inhibition assay.

Compounds	IC_50_ (μM)	Compound Group
Rutin	216.363 ± 4.4627	Flavonoids
Hesperidin	287.179 ± 3.0712	Flavonoids
Procyanidin B_2_	338.298 ± 12.7432	Tannins
Quercitrin	384.946 ± 5.2333	Flavonoids
Eriodictyol	634.116 ± 5.18442	Flavonoids
(−)-Epicatechin	650.370 ± 10.6953	Flavonoids
(+)-Catechin	660.377 ± 6.5982	Flavonoids
Caffeic Acid	796.218 ± 12.4298	Organic acids
Oseltamivir Acid	275.068 ± 4.4973	

## Supplementary Materials

Supplementary materials are available online.
