# Supplementary material for: Neuraminidase Inhibitory Activity and Constituent Characterization of Fagopyrum dibotrys
Source: Molecules. 2017 Nov 18;22(11):1998. doi: 10.3390/molecules22111998 (PMC6150301; doi:10.3390/molecules22111998)
Supplement: Supplementary file 1 [file molecules-22-01998-s001.pdf]

Article

Neuraminidase inhibitory activity and constituent characterization of Fagopyrum dibotrys.

Xiang Zhang[1], Yu Cao[1], Jinhua Li[1], Ailin Liu[2], Haibo Liu[1*], Linfang Huang*[,1]

[1]Institute of Medicinal Plant Development, Peking Union Medical College & Chinese Academy of Medical Sciences, Beijing 100193, China, [2]Institute of Materia Medica, Chinese Academy of Medical Sciences& Peking Union Medical College, Beijing, 100050, China

Table S1 The absolute values of INT on 8 main chemical compounds in *F. dibotrys*.

Compounds	INT(Kcal/mol)
rutin	149.599
hesperidin	210.412
procyanidin B2	170.195
quercitrin	124.383
eriodictyol	122.719
(-)-epicatechin	73.401
(+)-catechin	108.114
caffeic acid	111.820
